# A Congenital Choledochal Cyst in an Adolescent: A Unique Case Report and the Role Liver-Specific Contrast Agents in the Diagnosis of Challenging Cases

**DOI:** 10.7759/cureus.24384

**Published:** 2022-04-22

**Authors:** Rayane Issa, Sana Hatoum, Nadine Yazbeck, Lena Naffaa

**Affiliations:** 1 Department of Diagnostic, Molecular, and Interventional Radiology, Icahn School of Medicine at Mount Sinai, New York City, USA; 2 Department of Diagnostic Radiology, American University of Beirut Medical Center, Beirut, LBN; 3 Department of Pediatrics and Adolescent Medicine, American University of Beirut Medical Center, Beirut, LBN; 4 Department of Radiology, Nemours Children's Hospital, Orlando, USA

**Keywords:** gadolinium ethoxybenzyl dimeglumine, magnetic resonance cholangiopancreatography, doppler ultrasound, congenital abnormalities, choledochal cyst

## Abstract

Choledochal cysts are rare congenital cystic dilatations of the biliary tree. They are most commonly present in female infants and young children, and their pathology remains unclear. The triad of intermittent jaundice, abdominal mass, and pain is found only in a minority of patients. Diagnosis and delineation of accurate biliary anatomy are crucial for surgical planning. This is most often successfully achieved with ultrasound and magnetic resonance cholangiopancreatography. The definitive treatment is cyst excision which decreases the risk of biliary carcinoma. We present an unusual case of a choledochal cyst in an adolescent boy with a review of the literature and emphasis on multi-imaging modalities, including the role of liver-specific gadolinium contrast agents in challenging cases to confirm the diagnosis.

## Introduction

Choledochal cysts (CC) are rare congenital cystic dilations of the biliary tract [1,2]. They are more prevalent in East Asian populations and young children, being four times more common in females [2]. The exact etiology of CC remains unclear. The most accepted theory is the presence of an anomalous union of the pancreaticobiliary duct (AUPBD) [1-3].

The triad of intermittent jaundice, abdominal mass, and pain is found only in a minority of patients [1,2,4]. Adults tend to present with complications like cholangitis, choledocholithiasis, pancreatitis, and malignant transformation, whereas children are more likely to present with an abdominal mass and jaundice [5]. Double common bile duct (CBD), sclerosing cholangitis, congenital hepatic fibrosis, pancreatic cyst, and annular pancreas are some associated congenital anomalies. On the differential diagnoses, list are biliary lithiasis, primary sclerosing cholangitis, pancreatic pseudocyst, biliary papillomatosis, biliary atresia, and biliary hamartoma [2].

Choledochal cysts are predominantly benign but can be associated with serious complications, including malignant transformation, recurrent cholangitis, pancreatitis, and cholelithiasis [1,2].

We present an interesting clinical scenario of a choledochal cyst in an adolescent boy with a review of the literature. We discuss multi-imaging modalities and highlight the role of liver-specific gadolinium contrast agents.

## Case presentation

**Figure 1:**
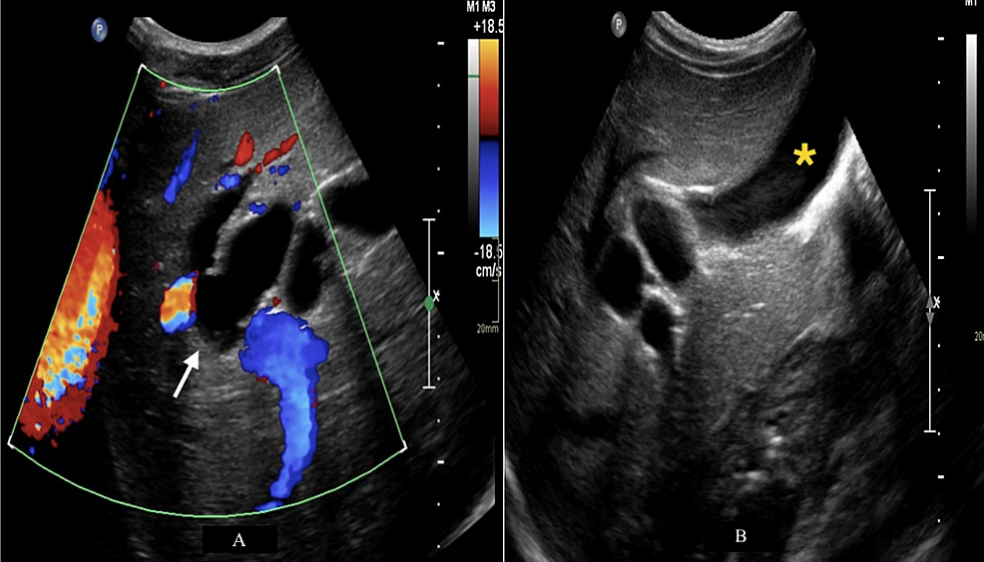
Axial ultrasound images of the liver and biliary tree Axial ultrasound images of the liver and biliary tree with doppler (A) and gray-scale (B) using a 5 MHz curvilinear probe showing cystic dilatation of intrahepatic bile ducts (A) (white arrow) with a normal gallbladder (B) (yellow asterisk). The common bile duct could not be visualized with certainty. The pancreatic duct (not shown) was of normal caliber.

A 16-year-old male with no significant past medical history presented to the emergency room for recurrent episodes of epigastric pain for three months radiating to the interscapular region with nausea and vomiting. The patient was not jaundiced, and the physical exam was unremarkable. Pertinent laboratory tests demonstrated elevated gamma-glutamyl transferase (657 U/L), alkaline phosphatase (493 U/L), alanine aminotransferase (233 U/L), and aspartate aminotransferase (79 U/L). Total and direct bilirubin were normal (0.9 mg/dL and 0.3 mg/dL, respectively), as well as lipase (50 U/L). Subsequently, an ultrasound was performed and showed cystic dilatation of intrahepatic bile ducts with normal gall bladder and pancreatic duct (Figures 1A, 1B).

**Figure 2:**
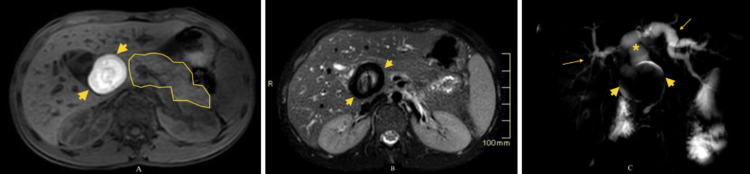
(A) Axial pre-contrast e-THRIVE (3T, TE 1.39, TR 3), (B) axial T2-weighted SPAIR (3T, TE 80, TR 1250), and (C) MRCP (3T, TE 800, TR 8000) Findings: A 5.0 x 4.0 cm round lesion in the region of the CBD (A, B, C) (short yellow arrows), in the vicinity of the duodenum, medial to the pancreatic head (A) (pancreas outlined in yellow). MRCP (C) shows the obstructing lesion (short yellow arrows) with dilated extra- (yellow asterisk) and intra-hepatic (long yellow arrows) bile ducts. e-THRIVE: enhanced T1W High-Resolution Isotropic Volume Examination, SPAIR: SPectral Attenuated Inversion Recovery, MRCP: magnetic resonance cholangiopancreatography, CBD: common bile duct

Further work-up with magnetic resonance cholangiopancreatography (MRCP) was performed to exclude the possibility of intrinsic (such as a stone) versus extrinsic obstruction. MRCP revealed, in addition to dilated intra- and extrahepatic hepatic ducts, a 5.0 x 4.0 cm round lesion in the topography of the common bile duct (CBD) in the vicinity of the duodenum, medial to the pancreatic head. The lesion showed an increased signal on T1 and T2 -weighted images and a thick low-intensity rim on T2-weighted images (Figures 2A-2C).

**Figure 3:**
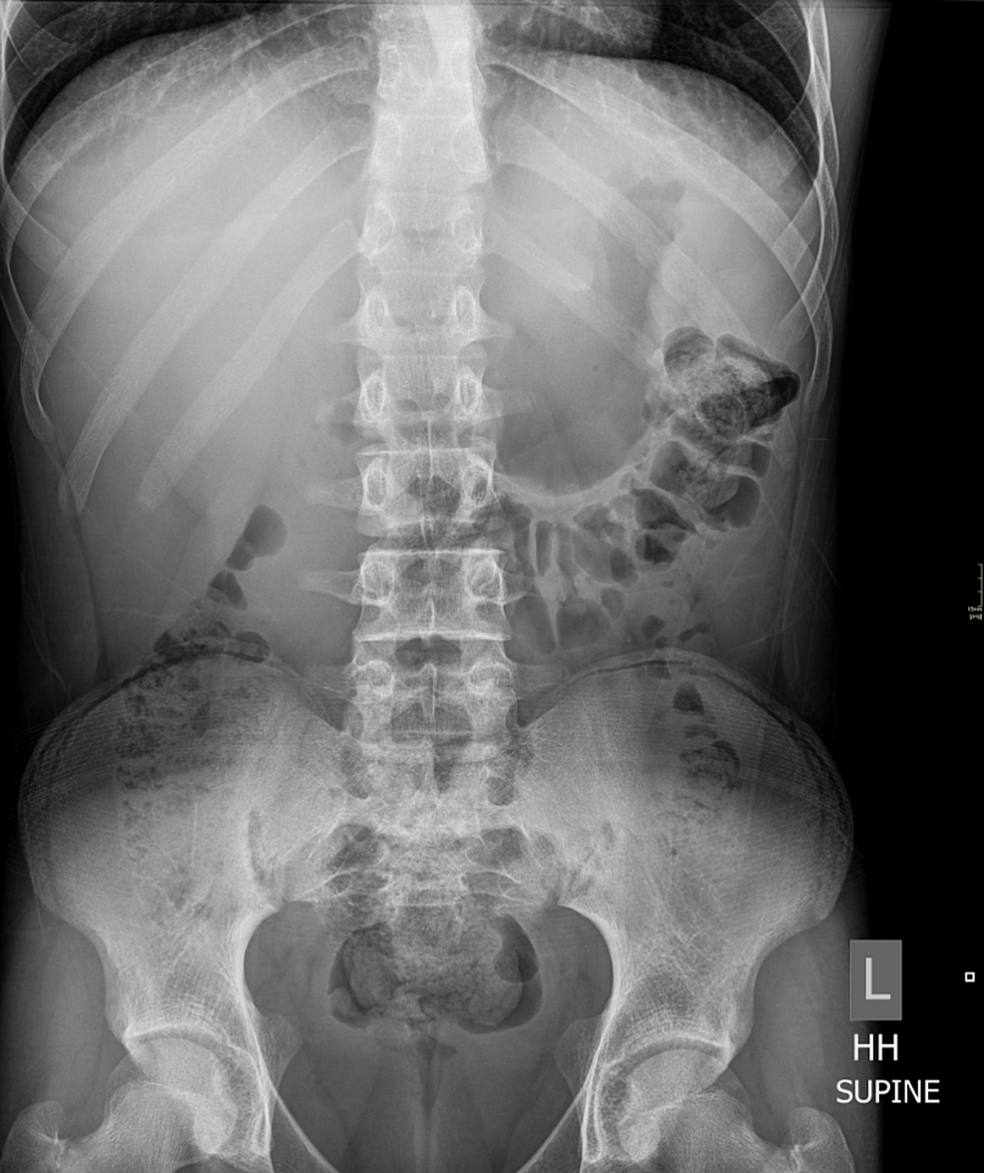
Conventional radiograph, supine abdomen, and pelvis (- 70 KV, 100 mAs) showing no calcifications

An abdominal radiograph (Figure 3) showed no calcifications in the topography of the previously detected anomaly.

**Figure 4:**
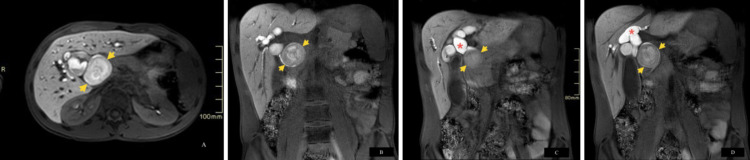
Axial (A) and coronal (B, C, D) dynamic e-THRIVE images obtained in hepatobiliary phase post administration of liver-specific contrast agent (Eovist) (3T, TE 1.38, TR 2.99) Findings: Contrast excretion within the biliary ducts (A, B) (red asterisks), outlining the previously described lesion (A, B, C, D) (short yellow arrows) within the CBD, confirming the diagnosis of choledochal cyst, type IVA. e-THRIVE: enhanced T1W High-Resolution Isotropic Volume Examination, CBD: common bile duct

The overall appearance favored the accumulation of mucin and proteinaceous material in a pre-existing CC, type IVA. However, the possibility of an extrinsic abnormality, such as duodenal diverticulum or old hematoma compressing the CBD, could not be completely excluded. Repeat imaging employing liver-specific hepatobiliary gadolinium contrast agent (Eovist) (Figures 4A-4D) showed contrast excretion within dilated intra- and extra-biliary ducts, outlining the entire contour of the previously described lesion and confirming its intraluminal nature and the diagnosis of CC, type IVA.

The CC was successfully removed surgically, without complications.

## Discussion

Choledochal cysts can be classified based on their imaging characteristics. The first imaging classification system was established in 1959 by Alonso-Lej et al. [2,6]. This classification system was then expanded by Todani et al. [7] and is now the most commonly adopted by clinicians (Table 1) [3,6].

**Table 1:** Classification of choledochal cysts CBD: common bile duct

Cysts type	Definition of cysts type	Cysts subtype	Definition of cysts subtype
Type I	Fusiform dilatations of the CBD which are further divided into three subtypes.	Type IA	Diffuse cystic dilatation of the extrahepatic biliary tree, sparing the intrahepatic ducts. The cystic duct arises from the dilated CBD.
		Type IB	Focal segmental dilatation which may occur anywhere along the CBD, but most commonly occurs distally. The cystic duct takes off the normal non-dilated part of the CBD.
		Type IC	Smooth fusiform dilatation of the entire extrahepatic biliary tree, extending from the pancreaticobiliary junction to the intrahepatic biliary tree.
Type II	True diverticula of the CBD with a narrow stalk.		
Type III	Focal intra-duodenal dilations of the CBD.		
Type IV	Multiple cystic formations of the biliary tree which are further divided into two subtypes according to the presence or absence of intrahepatic biliary ductal involvement.	Type IVA	Multiple intrahepatic and extrahepatic biliary dilatations.
		Type IVB	Extrahepatic biliary dilatation with a normal intrahepatic biliary tree, often compared to a "string of beads."
Type V (Caroli's disease)	Isolated cystic dilation of the intrahepatic biliary tree, sparing the extrahepatic biliary tree.		

Type V cysts must be differentiated from Caroli’s Syndrome, which occurs when type V CC are accompanied by congenital hepatic fibrosis [2].

The most commonly encountered types are I and IVA [4,6], and the latter was diagnosed in the presented case.

Selecting the appropriate imaging test is always more challenging in the pediatric population. Multiple points need to be taken into consideration, starting with avoiding ionizing radiation as much as possible. However, the radiograph was performed in our case to help exclude more common etiologies, including kidney stones, bowel obstruction, and bowel dilatation. In addition, young pediatric patients or older pediatric patients with special needs may be unable to tolerate long exams, hold still or hold their breath, and may require sedation and anesthesia. However, our case was a 16-year-old patient who was cooperative with the imaging procedures. Furthermore, the smaller size of the anatomic structures in the pediatric population poses challenges in identification and imaging, which results in the need for higher resolution imaging [8].

Ultrasound is usually performed as the first line of imaging. It is quick, non-invasive, non-radiating, and the least costly. However, it can be limited by the skill level of the users. It can demonstrate biliary ductal dilatation and can often help narrow the differential diagnosis, but it is least likely to provide a definite diagnosis, especially since visualization of the distal CBD may be limited by overlying bowel gas [8], as in our case. However, when it comes to biliary ductal abnormalities, the establishment of an accurate anatomy of the biliary ducts is crucial for surgical planning to avoid surgical and post-surgical complications.

Endoscopic retrograde cholangiopancreatography (ERCP) was traditionally the gold standard for the diagnosis and accurate classification of CC and associated anomalies of the biliary tree [4,8,9]. However, it is an invasive procedure that has been largely replaced by MRCP, a non-invasive tool with good overall accuracy in the detection and classification of CC due to excellent 3-dimensional mapping of both the biliary and the pancreatic ducts [4,8,10]. MRCP also reliably identifies APBDU, cholangiocarcinoma, and choledocholithiasis with concurrent CC [2]. The basic MRCP concept is based on heavily weighted T2 sequences with fat suppression, which will result in high signal intensity from slow-moving or static fluid with suppression of the signal from the surrounding tissues [8,11]. Thin slices are ideally acquired using the Half-Fourier Acquisition Single-shot Turbo spin Echo/Single Shot Fast Spin Echo (HASTE/SSFSE) sequence with multiple intensity projection (MIP) reconstructions [11].

Contrast agents are not usually necessary for diagnosis. However, in certain equivocal cases such as ours, the use of liver-specific contrast agents can be of important diagnostic value [12]. In liver magnetic resonance imaging (MRI), contrast agents are categorized into non-specific agents that distribute into the vascular and extravascular extracellular spaces, and liver-specific agents taken up by liver cells [13]. Initially, both non-specific and specific contrast agents behave the same [8,13]. Peak arterial enhancement typically occurs within 30 seconds and peak portal venous enhancement within 60 seconds [8] while accounting for cardiac output and age. However, after 60 seconds, liver-specific agents will be either taken up by Kupffer cells or by hepatocytes, which will then excrete them within the biliary system over the next 20-40 minutes [8,13], allowing for optimal visualization of intra- and extra-hepatic ductal anatomy. This is known as the hepatobiliary phase.

Gadolinium ethoxybenzyl dimeglumine (Gd-EOB-DTPA, Primovist in Europe, and Eovist in the USA) is the most commonly used FDA-approved liver-specific MRI contrast agent with up to 50% hepatobiliary excretion in the normal liver [8,13]. This is useful for the detection of bile duct leaks, the grading of bile duct obstruction, the differentiation between Caroli’s disease and peribiliary cysts, and the differentiation between parenchymal and small bile duct diseases [8,13]. The use of liver-specific contrast and dynamic imaging can also allow the visualization of bile reflux into the pancreatic duct and suggest pancreatic enzyme reflux into the bile duct in patients with AUPBD [14].

A disadvantage of this technique is poor or even absent Gd-EOB-DTPA-enhancement in patients with advanced diffuse parenchymal disease or bile duct obstruction [13]. Another disadvantage is the associated risk of nephrogenic systemic fibrosis (NSF). According to the American College of Radiology, Eovist is classified as a group III Gadolinium-based contrast agent, stating that these are “agents for which data remains limited regarding NSF risk, but for which few, if any, unconfounded cases of NSF have been reported.” As such, the use of Eovist remains unpreferred in patients with acute kidney injury, patients on dialysis of any kind, and patients with severe or end-stage chronic kidney disease (CKD) (CKD 4 or 5, estimated glomerular filtration rate < 30 mL/min/1.73 m2) without dialysis, unless there is no alternative, benefit clearly outweighs the risk, and risks were clearly discussed with the patient and the referring physician [15].

Total cyst excision is the definitive treatment of CC due to the risk of malignant transformation, which is most commonly associated with types I and IV cysts [2,16]. Complete resection decreases malignancy risk, which is crucial in the pediatric population with longer life expectancy [16]. It is generally well-tolerated [16], with postoperative morbidity and mortality being very low in children [2]. Complications most commonly occur with type IVA cysts and include intrahepatic stones and anastomotic stricture [2]. Early complications include anastomotic leak, postoperative bleeding, wound infection, acute pancreatitis, and pancreatic or biliary fistula [16]. Anastomotic stricture, cholangitis, hepatolithiasis, cirrhosis, and malignancy are some late complications [16].

## Conclusions

Choledochal cysts are rare and predominantly congenital in nature, but this case sheds light on the importance of keeping choledochal cysts among the differential diagnoses, even in older children. Diagnosis and delineation of accurate biliary anatomy are crucial for surgical planning. This is most often successfully achieved with ultrasound and MRCP. Liver-specific gadolinium-based contrast agents are reserved for the most challenging cases, especially in the pediatric population, where the anatomic structures may be much smaller than in adults. This technique is most likely to prove problem-solving, such as in our case, allowing prompt diagnosis and surgical resection.
